# Supplementary material for: Do Hatchery-Reared Southern Pygmy Perch (Nannoperca australis) Develop Effective Survival Behaviour in a Soft-Release Site?
Source: Animals (Basel). 2025 Sep 21;15(18):2754. doi: 10.3390/ani15182754 (PMC12466502; doi:10.3390/ani15182754)

Supplementary materials

Table S1: Latency to emerge from the refuge (mean, median, and IQR).

Latency to emerge from the refuge (s)	Mean	Median	IQR
**Juvenile**	92.2	34.0	120.0
**Hatchery**	141.0	57.5	260.0
**Soft Release**	73.3	1.0	70.8
**Wild**	101.0	1.0	124

Table S2: Percentage of fish demonstrating particular responses in the predator tests. Note that a fish may have demonstrated both an avoid and freeze response.

	**Avian predator**		**Fish predator**	
	**Avoid**	**Freeze**	**Avoid**	**Freeze**
**Juvenile**	54.80%	80.60%	67.70%	61.30%
**Hatchery**	66.70%	53.30%	56.70%	53.30%
**Soft release**	77.80%	77.80%	70.40%	66.70%
**Wild**	60.00%	80.00%	73.30%	60.00%

Table S3: Latency to food inspection (mean, median, and IQR) in the novel food test.

Latency to inspect food (s)	Mean	Median	IQR
**Juvenile**	1102.2	753.0	948.0
**Hatchery**	1741.1	1765.0	2180.0
**Soft Release**	1638.5	2163.5	1669.3
**Wild**	1229.8	999.0	1819.8

Table S4: Summary of fish assemblages from Sheepwash Creek (site of Wild fish) from surveys using fyke nets and bait traps deployed in 2019, 2022, 2023 and 2024.

Species	Number of fish
Carassius auratus	4
Galaxias oliros	173
Gambusia holbrooki	192
Nannoperca australis	273
Perca fluviatilis	4
Philypnodon grandiceps	17
**Total**	**663**

Figure S1: Collection points showing Soft Release fish collection (SR) from a man-made pond, and Wild fish collection (W) from a creek system.

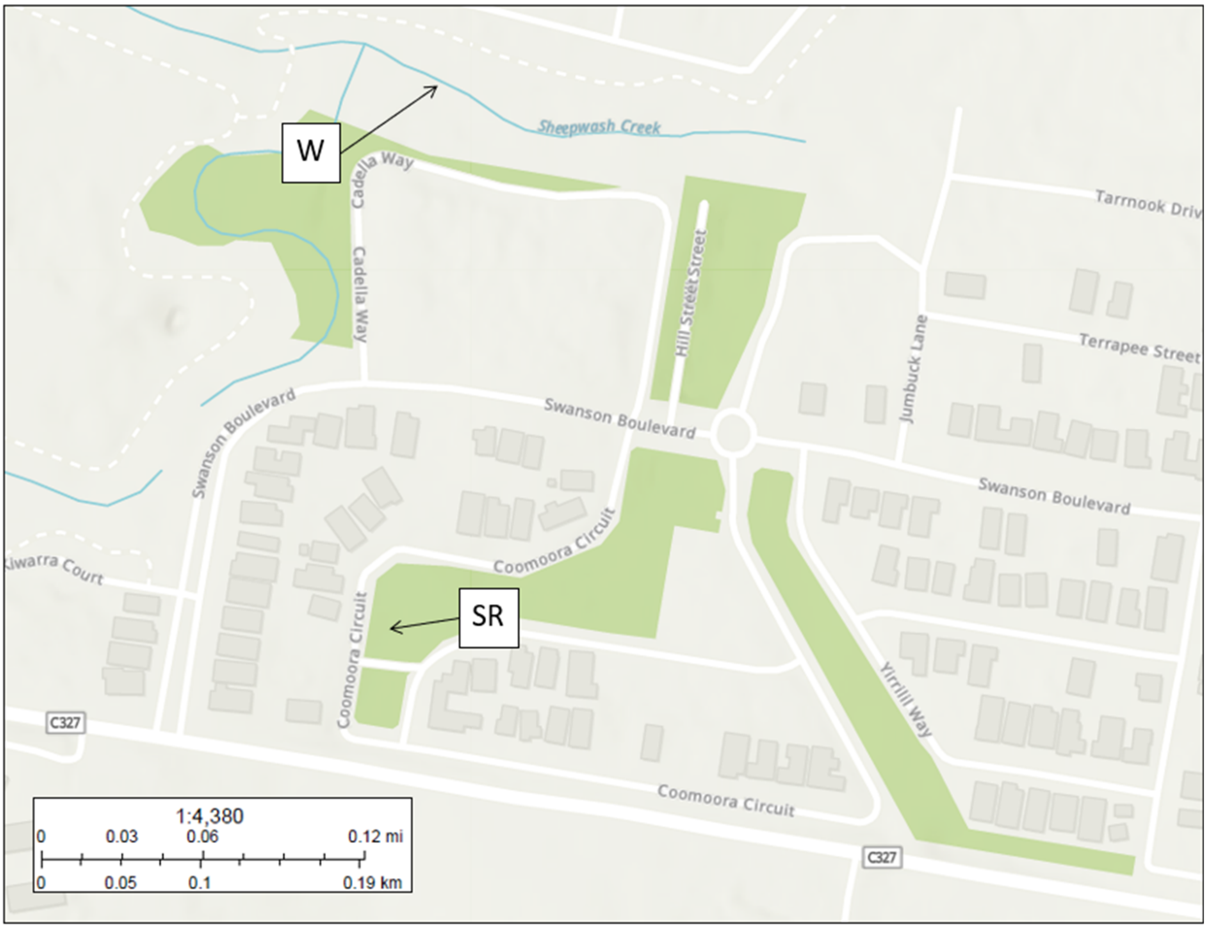

Figure S2: (a) Plus maze apparatus, showing a fish emerging from the refuge into A1 (left-most arm). (b) Habitat choice set up, showing (clockwise from left) empty, small rocks, plants, and large rocks; a SPP is being acclimated in the centre, and barriers between the centre and the habitats are in place. (c) Avian predator model on mechanical rotating stand.

(a)

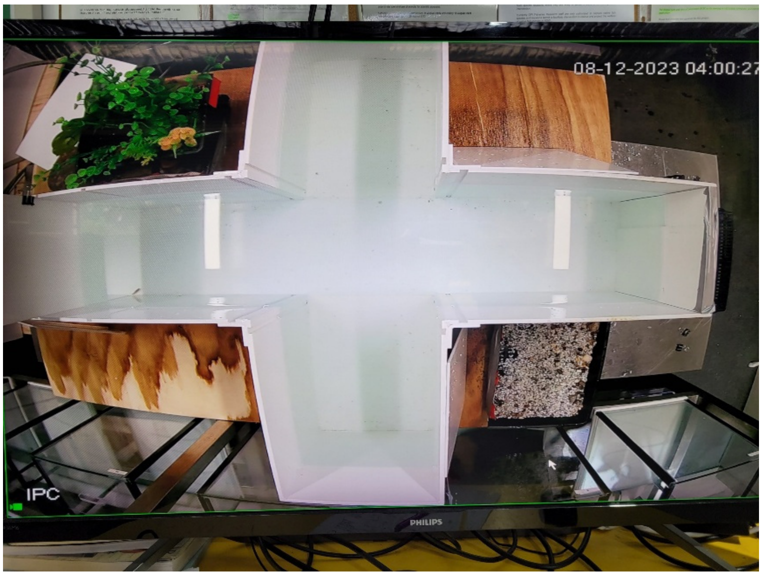

(b)

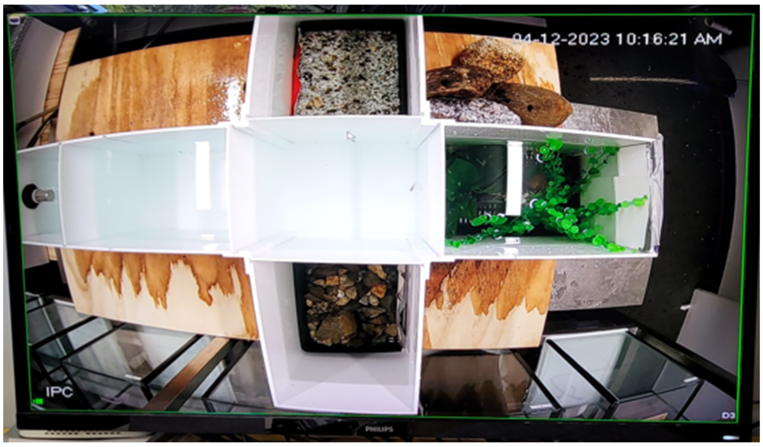

(c)

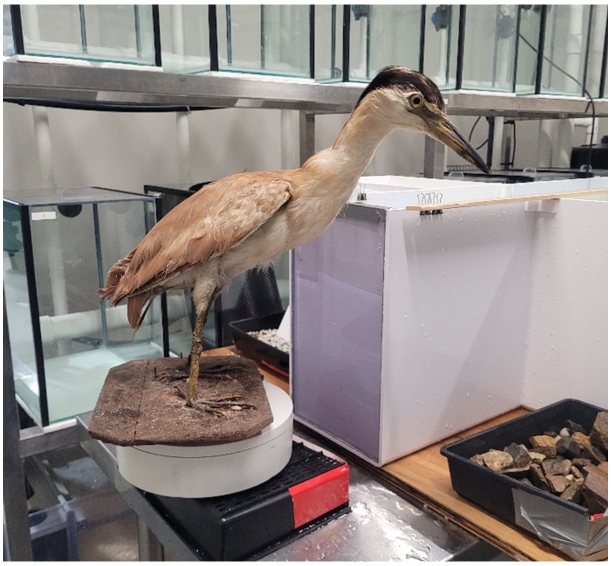

Figure S3: Latency to demonstrate a freeze response to the (a) model avian and (b) fish predator, for SPP that demonstrated a freeze response. Plots represent mean (line inside boxes) values, 25–75 percent quartiles (boxes), and range ("whiskers"). Outliers are represented with a black circle.

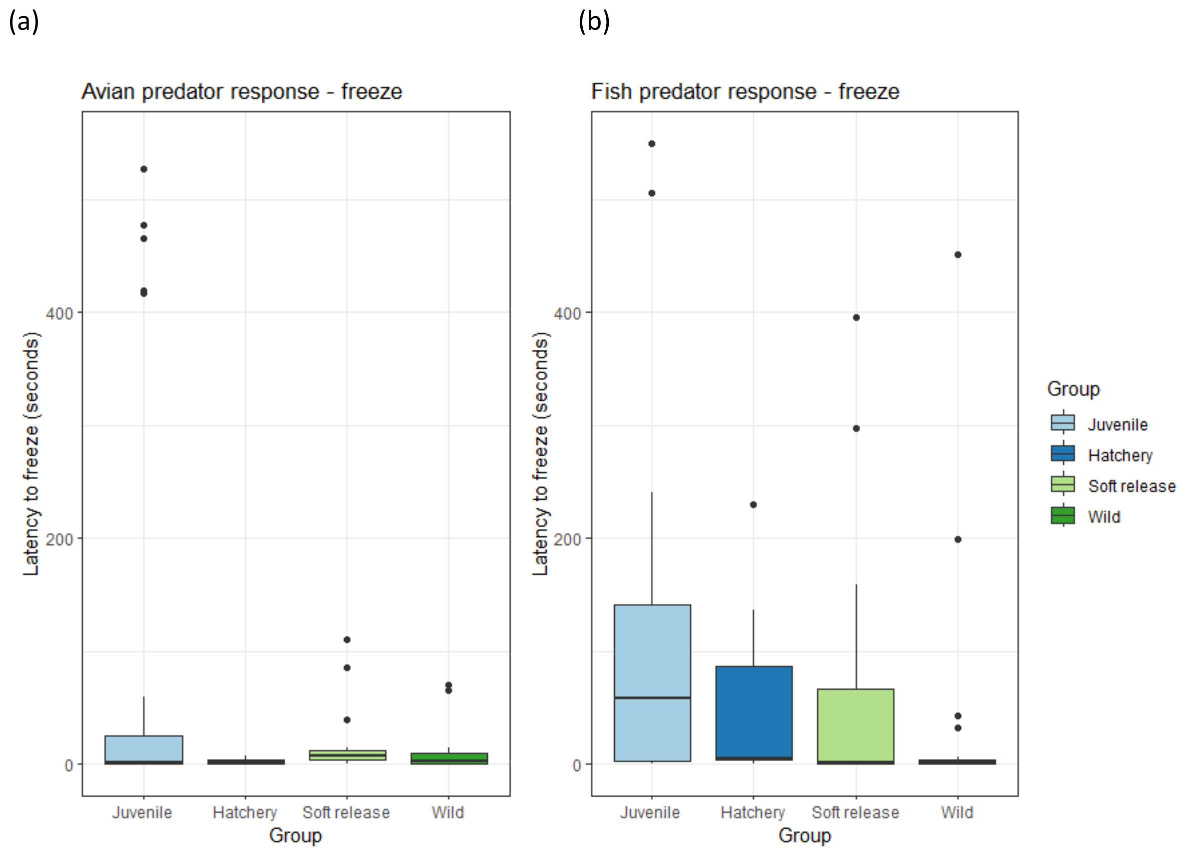

Figure S4: Colouration differences between SPP of same age and same genetic history, showing (a) Hatchery fish; and (b) Soft release fish following recapture.

(a)

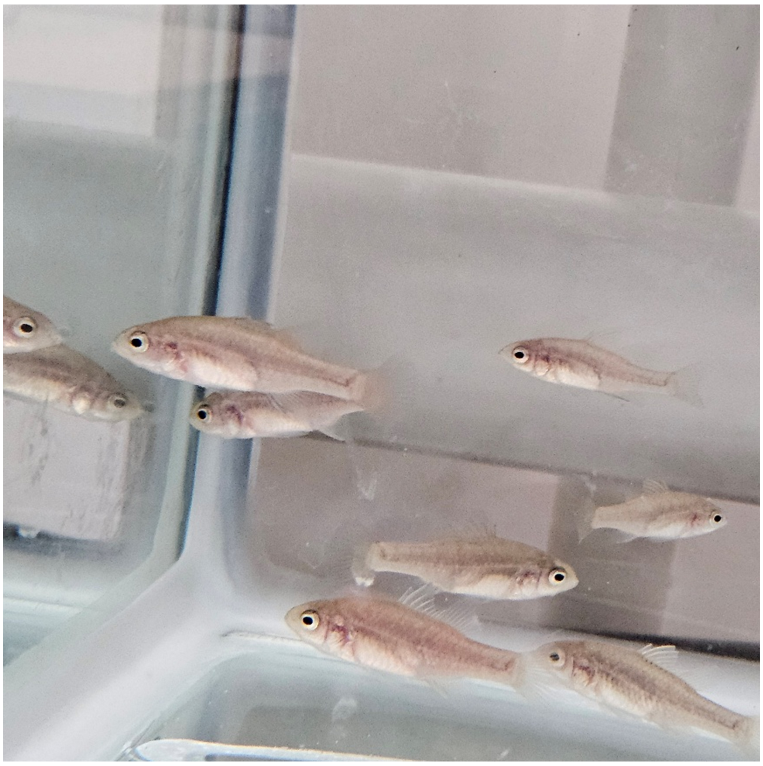

(b)

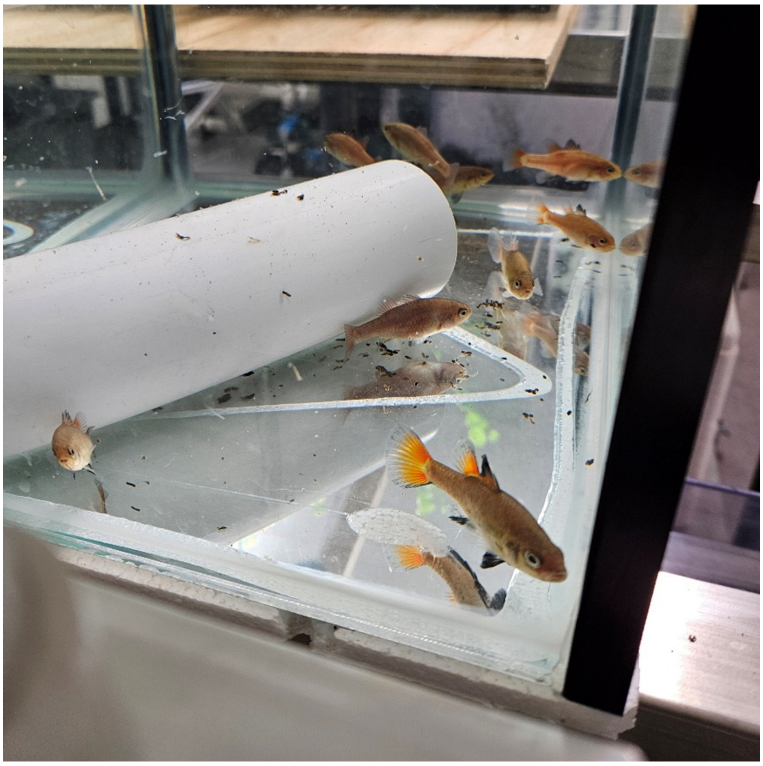

Supplement: Supplementary file 1 [file animals-15-02754-s001.zip › animals-3866268-supplementary.pdf]
